# Enhanced cutaneous wound healing in rats following topical delivery of insulin-loaded nanoparticles embedded in poly(vinyl alcohol)-borate hydrogels

**DOI:** 10.1007/s13346-018-0554-0

**Published:** 2018-07-03

**Authors:** Dalia H. Abdelkader, Murtaza M Tambuwala, Christopher A. Mitchell, Mohamed A. Osman, Sanaa A. El-Gizawy, Ahmed M. Faheem, Mohamed El-Tanani, Paul A. McCarron

**Affiliations:** 1 School of Pharmacy and Pharmaceutical Sciences, Saad Centre for Pharmacy and Diabetes, Ulster University, Cromore Road, Coleraine, Co., Londonderry, BT52 1SA UK; 2 Faculty of Pharmacy, Pharmaceutical Technology Department, Tanta University, Tanta, 31111 Egypt; 3 School of Biomedical Sciences, Ulster University, Cromore Road, Coleraine Co., Londonderry, BT52 1SA UK; 4 Sunderland Pharmacy School, University of Sunderland, Sunderland, SR1 3SD UK; 5 Institute of Cancer Therapeutics, University of Bradford, Bradford, West Yorkshire BD7 1DP UK

**Keywords:** Acute diabetes, Recombinant human insulin, PLGA nanoparticles, Topical delivery, Wound healing

## Abstract

Insulin plays an important role in the wound healing process, but its method of delivery to the wound bed and subsequent effect on rate of healing is less well investigated. In this study, we evaluated the therapeutic effectiveness of topical human insulin delivery using a nanoparticulate delivery system suspended in a structured hydrogel vehicle. Poly(lactide-co-glycolide) (PLGA) nanoparticles (NP) of 202.6 nm diameter and loaded with 33.86 μg insulin per milligram of polymer were formulated using a modified double-emulsion solvent evaporation technique and dispersed in a dilatant hydrogel (poly(vinyl alcohol)-borate). Importantly, this hydrogel formulation was used to achieve ultimate contact with the wound bed. A comparison of wound healing rates following local administration of insulin in the free and nanoencapsulated forms was performed in diabetic and healthy rats. In non-diabetic rats, there was no significant difference between healing observed in control and wounds treated with free insulin (*p* > 0.05), whereas treatment with insulin encapsulated within PLGA NP showed a significant difference (*p* < 0.001). In diabetic cohorts, both free insulin and nanoencapsulated insulin induced significant improvement in wound healing when compared to controls, with better percentage wound injury indices observed with the colloidal formulation. At day 10 of the experiment, the difference between percentage wound injury indices of insulin-PLGA NP and free insulin comparing to their controls were 29.15 and 12.16%, respectively. These results support strongly the potential of insulin-loaded colloidal carriers for improved wound healing when delivered using dilatant hydrogel formulations.

## Introduction

Diabetes is a multisystem disorder with a negative impact on wound restoration. This leads in many instances to the formation of chronic trauma and disruption of normal skin. Poorly controlled blood glucose levels are the primary cause, deterring the normal course of wound healing. Reductions in plasma fibronectin, pro-angiogenic growth factors and chemokines all delay the inflammatory response and deactivate proliferative fibroblasts, leading to apoptosis in the diabetic wound bed [1]. These pathological changes interfere with restoration of viable tissue that normally occurs during the well-recognised phases of normal wound healing [2]. Predictably, the importance of insulin in the wound healing progress has been known for many years when observations of differences between post-operative wound repair in diabetic and non-diabetic patients became apparent [3]. Topical application of insulin generally improves wound tensile strength and decreases healing time [4] and subcutaneous insulin depot increases the rate of musculopertoneal wound healing in rats [5].

The mechanism by which insulin enhances the rate of wound healing is not completely understood. Previous studies demonstrate that insulin improves tissue repair through stimulation of the PI3-K/AKT-Rac1 (phosphatidylinositol-3-kinase and protein kinase B-Ras-related C3 botulinum toxin substrate 1) migration pathway in keratinocytes [1, 6]. Furthermore, insulin receptor substrate 1 (IRS-1), PI3-K, AKT, glycogen synthase kinase 3 (GSK-3) and vasoactive endothelial growth factor (VEGF) expression in the skin of diabetic animals is elevated by topical insulin application, which confirms that insulin can stimulate a variety of cellular functions, such as cell differentiation and growth [2]. The topical approach is an immediately obvious delivery means and the findings of several studies demonstrate improved wound healing following the in vivo effect of topical insulin (solution, cream and alginate dressing) compared with control (no treatment) [7–10]. However, the beneficial effect of topically applied insulin in the free form is not always demonstrated. For example, Azevedo et al. stated that topical insulin cream does not modify the healing process in non-diabetic animals. They reported a non-significant difference in percentage wound area closure between non-diabetic rats treated with insulin-containing cream and placebo at day 14 post-wounding. Furthermore, given that some studies have applied insulin solution in a low-viscosity irrigation vehicle [9], then there is debate as to how effective absorption is, given the likelihood of poor residency time.

Our group has a particular interest in the use of novel hydrogel delivery systems that possess improved site retention at locations of disrupted skin. Results from our previous work highlight the importance of effective delivery and demonstrate that recombinant human insulin has an observable effect on the migration and proliferation of keratinocytes and fibroblasts [10]. These cells are known to play a pivotal role in the development of granulation tissue [2]. Importantly, our in vitro results demonstrate a significant improvement in cell migration when administered insulin is encapsulated within poly(lactide-co-glycolide) (PLGA) nanoparticles. This improvement is observed when compared to the proliferative effects resulting from insulin delivered in the free form [10]. Although insulin receptors are located on the plasma membrane, studies demonstrate their presence on many intracellular organelles. As free insulin is not able to readily cross the plasma membrane, the use of endocytosed insulin-loaded carriers could provide a novel means to bring about binding to intracellular insulin receptors [10]. Therefore, the first aim of this present study was to encapsulate insulin in a nanoparticulate carrier using a double-emulsion procedure and to verify a standard set of colloidal characteristics, such as size, charge, payload stability and loading efficiencies. The effect of adding poly(ethylene glycol), a commonly used hydrophilic, non-ionic and biocompatible polymer, to the primary organic emulsion phase was included in this part of the investigation. Poly(ethylene glycol) (PEG) is known to affect biological payload stability and cellular uptake, together with imparting beneficial effects on particle size and entrapment efficiency. The second aim of this study was to evaluate the in vivo effect of nanoencapsulated insulin. To achieve this, a novel poly(vinyl alcohol) (PVA)-borate hydrogel containing human insulin encapsulated in PLGA nanoparticles (NP) was developed and characterised. These hydrogels, which are based on cross-linked PVA-borate networks, are defined by interesting viscoelastic properties [11]. These properties make them an ideal and unique vehicle for topical delivery to both acute and chronic wounds and overcomes the poor residency time of low-viscosity vehicles, such as normal saline flushes [9]. The efficacies on wound healing in diabetic and healthy animal subjects were evaluated.

## Material and methods

### Materials

PVA (Mw = 31,000–50,000 Da; 87–89% hydrolysed), PEG flakes (Mw = 2000 Da), sodium tetrahydroxyborate decahydrate, insulin (recombinant human; dry powder), poly(D,L-lactide-co-glycolide) (acid terminated; lactide/glycolide 50:50; Mw = 24,000–38,000) and streptozotocin (STZ) were also purchased from Sigma-Aldrich Ltd., Poole, UK. Dulbecco’s phosphate-buffered saline (DPBS) and sodium hydroxide (NaOH) were purchased from Fisher Scientific, Loughborough, UK. A bicinchoninic acid assay (BCA) protein assay kit was purchased from Thermo Fisher Scientific, Pierce Biotechnology Inc., Rockford, USA. Trifluoroacetic acid (TFA), hydrochloric acid (HCl) solution, acetonitrile, dichloromethane (DCM) and methanol were of HPLC grade. All other reagents and solvents were of appropriate laboratory standard and used without further purification.

### Preparation of PVA-borate hydrogel loaded with free recombinant human insulin

PVA (24% *w*/*w*) and borate (5% *w*/*w*) stock solutions were mixed at 70–80 °C for approximately 1 h with periodic stirring. The final concentrations of PVA and borate were adjusted to 6 and 2% *w*/*w*, respectively, to produce a structured hydrogel, as described previously [9]. Recombinant human insulin, dissolved in a 1:1 ratio (pH 1.5–2.0) of 0.1 M HCl and 2.5% *w*/*w* PVA, was added to the PVA-borate formulation during cooling in an ice bath, with continuous stirring. Hydrogels were stored at 4.0 °C for 48 h prior to further analysis.

### Preparation of insulin-PLGA nanoparticles embedded in PVA-borate hydrogel

Insulin-loaded NP were prepared using a modified double-emulsion, solvent evaporation technique. Briefly, an aqueous insulin solution (0.10 ml, 5%) was added to a DCM phase (4.00 ml) containing PLGA (5%) and PEG (0 or 5%). This primary emulsion was homogenised for 2 min at 1000 rpm (Ultra-Turrax® T10 Basic Disperser, IKA® Works) before dropwise addition to an external aqueous phase (50.0 ml) containing 1.25% *w*/*v* PVA [12], with continuous stirring for 6 min at 10,000 rpm (model L5M-A Silverson Ltd., UK). NP were stirred to remove DCM, then collected by centrifugation at 4 °C. After washing, the pellet was frozen at − 20 °C for 6 h and lyophilised (4.5 Plus, Labconco Ltd., USA) for 48 h. The lyophilised NP pellet was dispersed in deionised water and added to a blank PVA-borate hydrogel, as described in section ‘Preparation of PVA-borate hydrogel loaded with free recombinant human insulin’.

### NP characterisation

Surface charge (zeta potential, mV) was determined by measuring electrophoretic mobility. Particle size (diameter, nm) and polydispersity index were determined by photon correlation spectrometry (ZetaSizer Nano series, Malvern Instruments, Worcestershire, UK). Measurements were performed in triplicate at 25 °C. NP in a lyophilised form or embedded in PVA-borate hydrogels were examined for surface morphology using scanning electron microscopy (SEM) (Zeiss, Oberkochen, Germany).

### HPLC analysis

Recombinant human insulin was analysed using reversed-phase HPLC (Shimadzu Corporation, Kyoto, Japan) on a Luna® C18 column (5 μm, 150 × 4.6 mm, Phenomenex, CA, USA). The mobile phase was a binary mixture of 0.1% trifluoroacetic acid in water and 0.1% trifluoroacetic acid in acetonitrile [13]. Gradient elution was performed by increasing acetonitrile concentration from 10 to 35% over a 15-min period at *λ*_max_ 210 nm with a flow rate of 1.1 ml min^−1^.

### Determination of insulin loading and entrapment efficiency

The amount of encapsulated insulin within the NP matrix was analysed using a BCA assay following digestion of lyophilised NP (15 mg) by incubation with 1.0 M NaOH for 2 h and subsequent neutralisation with 1.0 M HCl (direct method) [14]. Drug loading (DL) was calculated as a ratio of mass of drug in the NP to the mass of NP. Direct entrapment efficiency (EE%) was expressed as a ratio of mass of the drug in NP to mass of the drug initially added during the manufacturing process [15]. An indirect method was also used to assess entrapment efficiency, calculated from insulin concentrations determined in the supernatant using the RP-HPLC method described in section ‘HPLC analysis’.

### In vitro drug stability—gel electrophoresis (SDS-PAGE)

Human insulin (control), a protein ladder (See Blue® Plus2 Pre-stained Protein Standard, Novex™ Thermo Fisher Scientific, UK), insulin released from both NP and PVA-borate hydrogels were placed in the wells of a NUPAGE® Bis-Tris 12% gel (Invitrogen, Thermo Fisher Scientific, UK) using a mini-cell electrophoresis system (X-cell Surelock™, Invitrogen, Thermo Fisher Scientific, UK). Peptide samples (10 μl) were vortexed with 2.0 μl Laemmli buffer (60 mM Tris-Cl pH 6.8, 2% SDS, 10% glycerol, 5% β-mercaptoethanol and 0.01% bromophenol blue) and heated at 100 °C for 10 min. Electrophoresis was run at 200 V (~ 100 mA) until samples reached the bottom of the gel. A fixative solution of Coomassie blue dye was used to stain the gel followed by addition of a destaining solution of methanol/acetic acid/water (5:4:1 *v*/v), which visualised the peptide bands. GelDoc-It™ (UVP, Cambridge, UK) was used to photograph the gel and record band positions.

### Rheological analysis

Relaxation time (*r*_*c*_), which measures how quickly the cross-linked network relaxes from a deformation after an applied shear [16], was determined using oscillatory rheometry, according to methods described previously [9]. The same technique was used to determine cross-over modulus (*G*_c_), which is taken as the intersection point between storage modulus (*G*′) and loss modulus (*G*″) when the hydrogel is under an oscillatory shear. To complete the mechanical analysis, the shear stress required to start flow after the destruction of the cross-linked hydrogel network [17], known as yield stress (*σ*), was also evaluated. All statistical analyses were carried out using rSpace® software (Kinexus version 1.2, UK).

### In vivo study

#### Animals

This research protocol was approved by Ulster University’s Animal Ethics Committee (Approval number: *DA 1/15-PIL 1601*) in accordance with the UK Animals (Scientific Procedures) Act of 1986. Sixteen male Sprague-Dawley rats (12 weeks old) weighing 300–350 g were used. All rats were kept in plastic cages and fed with standard laboratory pellets with potable water ad libitum until the day of the experiment. After wound creation, the rats were housed individually in separate cages.

#### Induction of type I diabetes

Diabetes was induced by injection of a single dose of intraperitoneal streptozotocin (STZ) at a dose of 50–60 mg kg^−1^ body weight in 0.1 M sodium citrate buffer at pH 4.5 [18]. Animals were fasted for 6 h prior to injection. Solutions were prepared freshly and injected immediately. All rats were checked for symptoms of polydipsia, polyuria and weight loss. A blood glucose measurement was performed 48 h after STZ injection [19]. Blood was drawn from the tail vein and the glucose level determined using a glucometer (Contour®, Bayer HealthCare LLC, US). Rats with blood glucose levels > 250 mg dl^−1^ were considered to be in a diabetic state. Rats in the non-diabetic group were injected with a single dose of intraperitoneal saline.

#### Experimental groups

Animals were randomly assigned to either diabetic or non-diabetic groups. This study was conducted using four groups of male Sprague-Dawley rats, with each group comprising four subjects. The assigned groups were (i) non-diabetic rats receiving topical free insulin loaded in a PVA-borate hydrogel (non-DB-H); (ii) non-diabetic rats receiving topical insulin-loaded NP (F2, see Table 1) embedded in a PVA-borate hydrogel (non-DB-NP); (iii) diabetic rats receiving topical free insulin loaded in a PVA-borate hydrogel (DB-H) and (iv) diabetic rats receiving topical insulin-loaded NP (F2) embedded in a PVA-borate hydrogel (DB-NP).

**Table 1:** Effect of PEG content on size, PDI, charge and drug entrapment in insulin-loaded PLGA NP

Formula code	PEG content (%)	*Z* average (nm)	PDI	Zeta potential (mV)	Drug loading (μg/mg NP)	Direct EE^a^ (%)	Indirect EE^b^ (%)
F1	0	297.8 ± 18.8	0.15 ± 0.02	− 3.94 ± 0.02	28.47 ± 5.35	56.9 ± 10.7	69.1 ± 0.6
F2	5	202.6 ± 20.6	0.38 ± 0.06	− 5.70 ± 0.17	33.86 ± 2.71	67.7 ± 5.4	69.5 ± 3.3

Data represent mean ± SD of three replicates. NP composition was insulin (5% *w*/*w*), PLGA content (2.5% *w*/*v*) and PEG 2 kDa

^a^Direct entrapment efficiency (EE) measured by BCA

^b^Indirect EE measured by HPLC

#### Wound model and treatment

An evaluation of the healing process was carried out by creating wounds 3 days after STZ or saline administration. On each animal subject, two circular, full-thickness excision wounds, 6.0 mm in diameter, were created with a punch biopsy tool on the dorsal thoracic area [20]. Rats were anaesthetised with ketamine (10 mg kg^−1^) and xylapan (0.3 mg kg^−1^) intraperitoneally, then the dorsal hair of the rats was shaved with an electrical clipper. After cleansing with depilatory cream, ketoprofen solution (5.0 mg kg^−1^) was injected subcutaneously for pain management, prior to surgical incisions. The day of wounding day was coded as day 0. After the excisions, a silicone splint was placed over each wound using an adhesive material to ensure positioning. PVA-borate hydrogel (0.5 g), containing 5.2 × 10^−3^ μM of recombinant human insulin (free or entrapped in PLGA NP-F2) [8], was applied to one wound and a saline control was applied in juxtaposition to the other. Wounds were then covered with an occlusive dressing.

#### Wound area closure measurements

Wound areas were measured daily and used to calculate a percentage wound injury index (Eq. 1). This index provided a numerical assessment of the decrease in wound area with respect to time. Thus, a percentage wound injury index of 100% at a particular time point would indicate no healing, whilst approaching 0% wound indicate complete closure of the wound. After induction of general anaesthesia using 5% isoflurane via an induction chamber and maintaining (via a nose cone) at 1–2% with O_2_, the occlusive dressing was peeled away with forceps. Surgical callipers were used to measure the wound diameter by taking the average of three measurements along Cartesian coordinates. PVA-borate hydrogel formulations and the saline control were re-applied at this point. A sterile, transparent, occlusive dressing was used to cover the wounds and the animals kept warm until fully recovered.

1 $$ \%\mathrm{wound}\ \mathrm{injury}\ \mathrm{index}=\frac{\mathrm{wound}\ \mathrm{area}\mathrm{at}\mathrm{time}t}{\mathrm{wound}\ \mathrm{area}\mathrm{at}\mathrm{time}\ \mathrm{zero}}.100 $$

#### Termination of the experiment

At the end of the experiment (days 12 and 16 for non-diabetic and diabetic groups, receptively), the rats were anaesthetised using isoflurane at 3–4%, and wound samples were harvested and incubated at 4 °C overnight, in 4% paraformaldehyde dissolved in phosphate-buffered saline (PBS), for further histological analysis. All animals were euthanised using the approved method of CO_2_ inhalation.

#### Histological examination

Skin tissue samples were fixed in 10% formaldehyde and processed appropriately for embedding in paraffin wax. After dehydration in an ascending series of ethanol solutions, tissue samples were cleared in toluene. Paraffin wax-embedded sections 4–6-μm thick were stained with haematoxylin and eosin to identify pathohistological changes during wound healing [21].

### Statistical analysis

Statistical analysis was performed using Prism 5 (Graph-Pad Software). A one-way ANOVA, followed by a pairwise comparison post hoc test, was conducted wherever appropriate. The significance level for rejecting the null hypothesis was 5% (*p* < 0.05). The effect of insulin addition (free or encapsulated in NP) on the mechanical properties of PVA-borate hydrogel was compared to a blank hydrogel formulation using Student’s *t* test.

## Results and discussion

### Insulin stability and NP characterisation

In this study, the delivery of insulin using a colloid carrier to newly excised wounds was studied. Of particular interest was the effect of insulin administration in its free and encapsulated forms on the rate of wound healing. Topical and transdermal applications of NP have been described extensively elsewhere, with reports detailing certain advantages when compared to other drug delivery systems. Colloidal carriers enhance the solubility of hydrophobic drugs, provide sustained and controlled release of encapsulated drugs, increase the stability of therapeutic agents by encapsulating within polymeric matrix and achieve site-specific delivery using vascular and cellular uptake mechanisms. Drug-loaded NP accumulate in hair follicles and facilitate the penetration of drug molecules through the superficial layers of the stratum corneum, followed by drug release into the deeper layers of the skin [22]. However, from a formulation perspective, the stability of encapsulated payloads is a concern, especially if it is peptide in nature and an emulsion-based procedure is used during manufacture.

**Fig 1:**
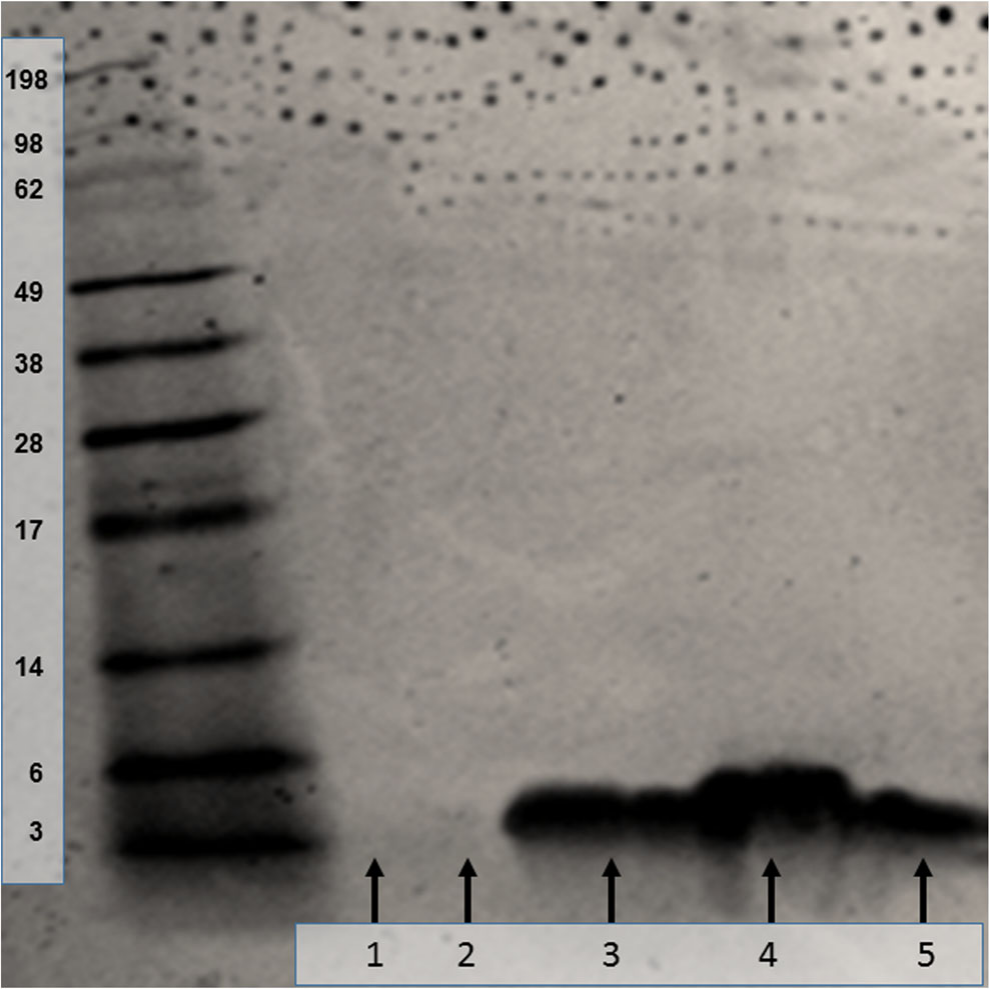
In vitro stability of human insulin released from NP and PVA-borate hydrogel as assessed using SDS-PAGE. Ladders indicate molecular weight in kilodalton. Lane 1 = blank NP. Lane 2 = blank PVA-borate hydrogel. Lane 3 = insulin released from NP. Lane 4 = control insulin. Lane 5 = insulin released from PVA-borate hydrogel

Emulsion-based encapsulation described in this work employed a non-aqueous phase (DCM), which is known to affect peptide stability during this type of procedure. This poor stability is attributed to interfacial effects separating the emulsion phases. It has also been suggested that high rates of shear produced during homogenisation of primary and secondary emulsion phases lead to three-dimensional alternation in peptide structure [23]. Given these concerns and the possibility of poor insulin stability, PEG addition was used during this work, which is a common approach to overcome interfacial aggregation. SDS-PAGE data (Fig. 1) showed the position of insulin bands obtained from (i) standard control, (ii) insulin released from NP, (iii) insulin released from PVA-borate hydrogel, (iv) placebo NP sample and (v) placebo hydrogel sample with no insulin loading. Comparisons with the protein ladder calibration showed that the insulin standard, insulin released from NP and free insulin released from a PVA-borate hydrogel formulation all have an approximate molecular weight of 6.00 kDa. This confirmed that human insulin entrapped in both a PLGA polymeric matrix and PVA-borate hydrogel did not aggregate.

PEG is a hydrophilic, non-ionic and biocompatible polymer. Its utilisation during NP manufacturing has beneficial effects, not only on stability but also on physical parameters, such as particle size and entrapment efficiency. PEG is able to rearrange the association of PLGA chains leading to a smaller mean diameter [24]. It is also a common moiety used as a surface modifier added to NP via several different methods, such as covalent binding, direct addition during NP fabrication or surface adsorption. The data in Table 1 show that addition of PEG, as used in this work, had a significant effect (*p* < 0.05) on particle size. The presence of PEG chains rearranges the structure of the PLGA polymeric matrix during the formation of NP, leading to a decrease in the particle size, as described by [24].

Zeta potential has an important bearing on nanoparticulate stability. When particulate charge changes from neutrality, electrostatic repulsion prevents unwanted aggregation. Addition of a surface modifying molecule, such as PEG, has been shown to impart a small, but nonetheless decisive, increase in negativity, which has the desired improvement in stability [25]. During NP formulation in this work, PEG was added to the PLGA-rich organic phase, creating an eventual deposition of peripheral PEG on the NP surface, which reduces protein adsorption, delays degradation and increases stability [25]. As shown in Table 1, the zeta potential values of F1 and F2 were not significantly different, but F2 was more negative.

There is much published data attempting to explain the effect of interfacial molecules, such as PEG, on the drug loading in NP produced using the double-emulsion method. In particular, emphasis focuses on preventing payload escape to the external aqueous phase, where drug is lost and loading is diminished [24]. A significant increase in DL and direct EE (%) was determined after the addition of PEG (*p* < 0.05) (Table 1). During the double-emulsion-based nanoencapsulation process, it is feasible that PEG chains were distributed at the interface layer between the insulin-containing internal phase and the organic phase. This effect prevents drug migrating towards the external aqueous phase, which might explain the higher encapsulation efficiencies [26]. Importantly, the choice of method used to measure entrapment efficiency had a bearing on the estimate of entrapped drug. Determination of EE (%) using direct and indirect methods resulted in higher values when the indirect EE method was compared to the direct EE (*p* < 0.05) method for NP formulation (F1) containing no PEG (Table 1). The indirect method for determination of EE (%) depends on detecting drug concentration in the supernatant and is, therefore, not an accurate measure of particulate content. Indeed, further processing, such as washing and centrifugation, removes loosely bound drug and so a preliminary analysis of the supernatant immediately following nanoprecipitate formation may be an overestimation. As seen in Table 1, the differences between both methods after PEG addition were not significant. This suggests that the insulin is firmly entrapped within the NP and not loosely bound to nanoparticulate surface.

**Fig. 2:**
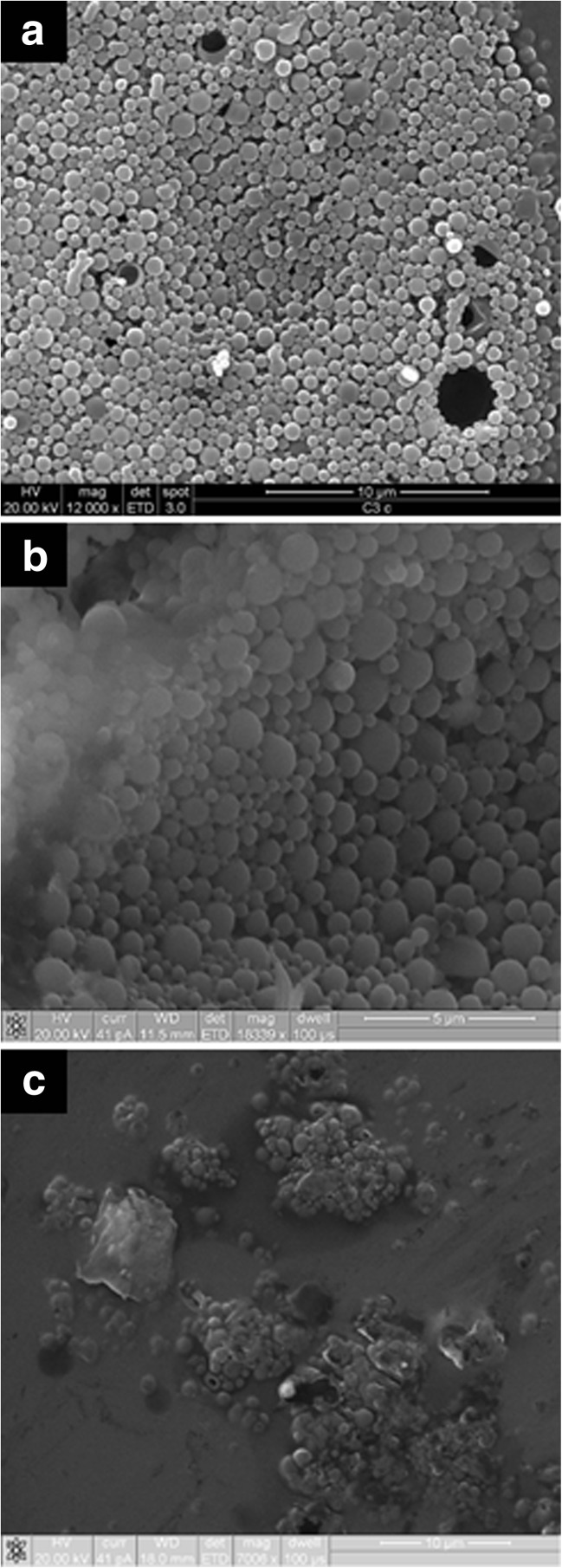
Scanning electron micrographs of insulin-loaded PLGA NP (F2) observed under low magnification (**a**), at higher magnification (**b**) and embedded in a PVA-borate hydrogel (**c**)

Information in scanning electron micrographs (Fig. 2a, b) showed that NP had a spherical morphology with a narrow size distribution. This agrees with polydispersity index (PDI) values presented in Table 1, indicating a mono-disperse population. Interestingly, the addition of PEG caused this distribution to become more spread. Visualising NP in the PVA hydrogel was a difficult procedure and SEM of these samples were aggravated by background artefacts, presumably due to desiccated hydrogel sheets. However, NP are clearly discernible in these structures (Fig. 2c).

### Viscoelastic studies

Although a detailed rheological analysis following addition of either free insulin or insulin-loaded NP to the PVA-borate hydrogel is beyond the scope of this study, a preliminary assessment is shown in Table 2. Significant changes (*p* < 0.05) in relaxation time and yield stress were noted. It is important to stress the function undertaken by PVA-borate hydrogels in this work in delivering insulin, either free or encapsulated, to the wound surface. These novel hydrogels predominantly exhibit elastic properties (storage modulus *G*′) when a force of high frequency is applied. This provides them with the structural integrity required to be moulded into any shape. This also permits the removal of the delivery system completely intact from the wound bed post treatment. When left to rest within the wound bed viscous properties (loss modulus *G*″) dominate. This results in the relaxation of the cross-linked matrix facilitating movement of the gel. The material will flow like a viscous liquid allowing the entirely of the wound to become filled, thereby maximising drug absorption [9].

**Table 2:** Effect of insulin addition on mechanical properties^*^ of PVA-borate hydrogels at room temperature (25 °C)

Formulation^a^	Crossover moduli *G*_*c*_ (Pa)	Relaxation time *r*_*c*_ (s)	Yield stress σ (Pas)
Placebo hydrogel	4593.67 ± 138.54	1.83 ± 0.01	402.33 ± 9.11
Free insulin hydrogel	3819.30 ± 77.36	1.62 ± 0.03	295.73 ± 20.05
Insulin NP embedded in hydrogel	4442.00 ± 128.35	1.69 ± 0.02	298.47 ± 20.01

^*^Data represent mean ± SD of three replicates

^a^Hydrogel composition was PVA (6% *w*/*w*) and borate (2.0% *w*/*v*)

The data in Table 2 show that the hydrogel structure is responsive to addition of the active agents and in particular, the addition of insulin in free form. The density of cross-linking between PVA chains and borate ions is the principle factor affecting the rheological characteristics of PVA-borate systems [27]. It can be concluded from the cross-over moduli that there is an interaction occurring between free insulin and the PVA-borate, leading to decreased cross-linking and a possible loss of elastic properties. This would indicate the formulation is more difficult to extract from the wound as it becomes more fluid [9]. The data in Table 2 indicate further that the same effect on crossover modulus is not observed in NP-loaded hydrogels. Clearly, encapsulation of insulin is averting its influence on cross-link density and is a favourable formulation strategy.

### Preliminary in vivo verification

**Fig. 3:**
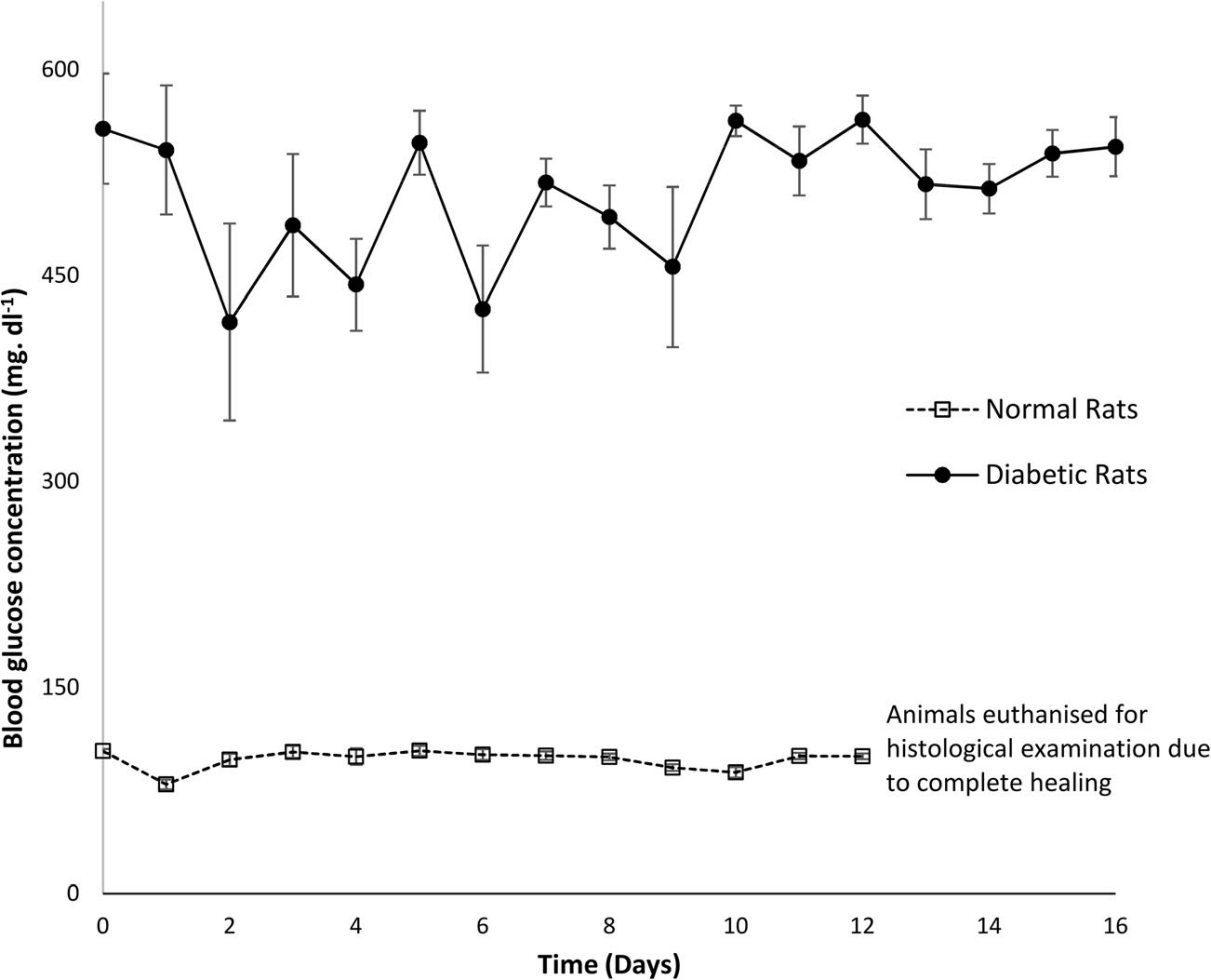
Average serum glucose concentration in diabetic and non-diabetic rats over the duration of the study period. Results are mean ± SEM (*n* = 8)

**Fig. 4:**
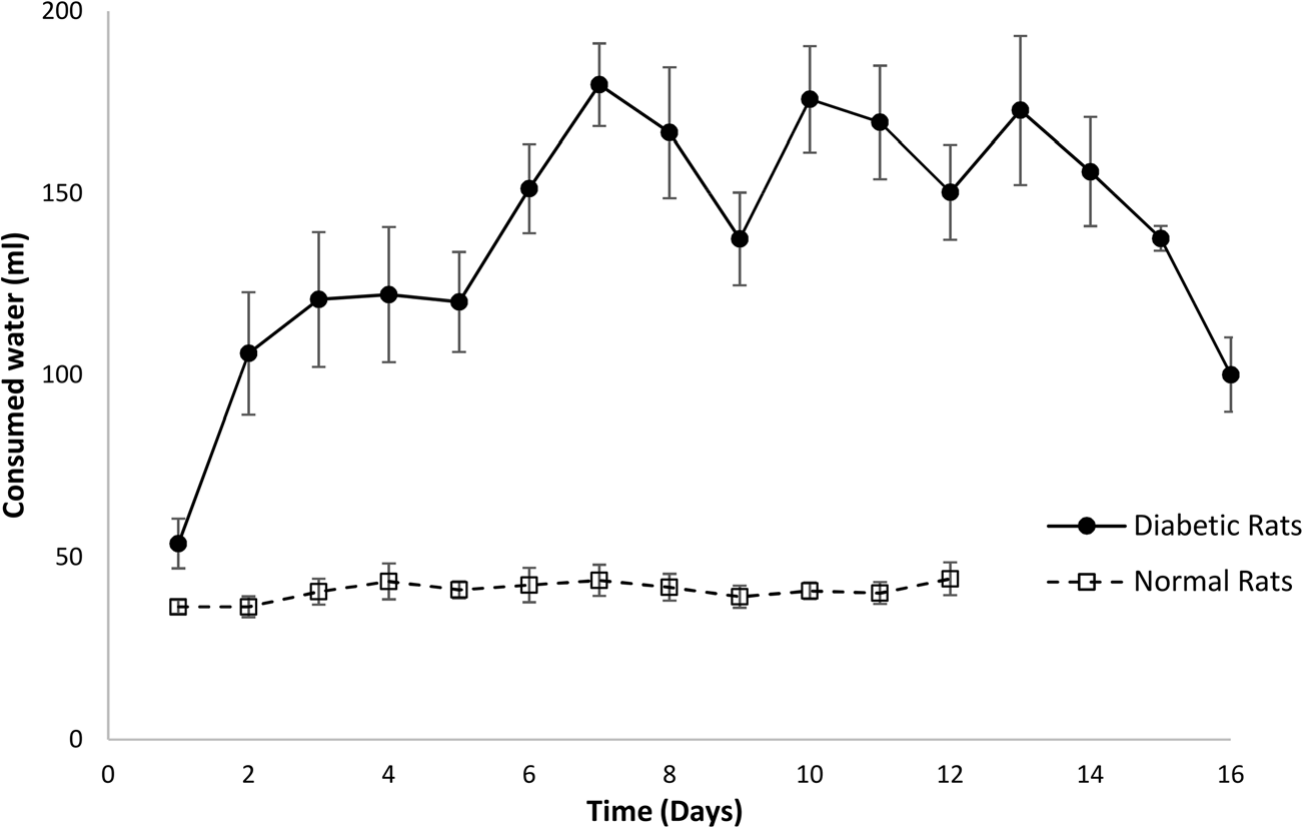
Average of daily water consumption of eight diabetic and normal rats over a period of 16 days. Non-diabetic animals were euthanised at day 12 for histological examination due to complete healing. Results are mean ± SEM (*n* = 8)

All rats that received STZ injection displayed symptomatic markers of diabetes, such as polydipsia, polyuria and weight loss. Induction of diabetes was confirmed by a blood glucose concentration ≥ 300 mg dl^−1^ measured 48 h after STZ injection in all animals in the diabetic group. All non-diabetic animals had blood glucose concentration < 150 mg dl^−1^. The mean pre-operative glucose concentration was 575.25 mg dl^−1^ for the diabetic and 103.83 mg dl^−1^ for the non-diabetic rats. It was found that blood glucose concentration stayed high throughout the study period for the diabetic animals (Fig. 3). Table 3 shows that the average glucose level of diabetic and non-diabetic groups was 509.38 and 97.64 mg dl^−1^, respectively, throughout the healing process. The daily water consumption was significantly higher (*p* < 0.01) for the diabetic group when compared to the non-diabetic rats, as shown in Fig. 4. Increased water consumption is a direct result of STZ-induced diabetes, with subsequent dehydration. Frequent urination and increased water intake are commonly observed in diabetic rats [28].

**Table 3:** Average body weight, glycaemia level and water consumption during healing process

State of rat	Weight (g)	Blood glucose (mg dl^−1^)	Water consumption (ml)
Normal (*n* = 8)	329.06 ± 4.52	97.64 ± 7.09	40.84 ± 2.54
Diabetic (*n* = 8)	308.30 ± 6.45	509.38 ± 43.95	138.79 ± 26.12

**Fig. 5:**
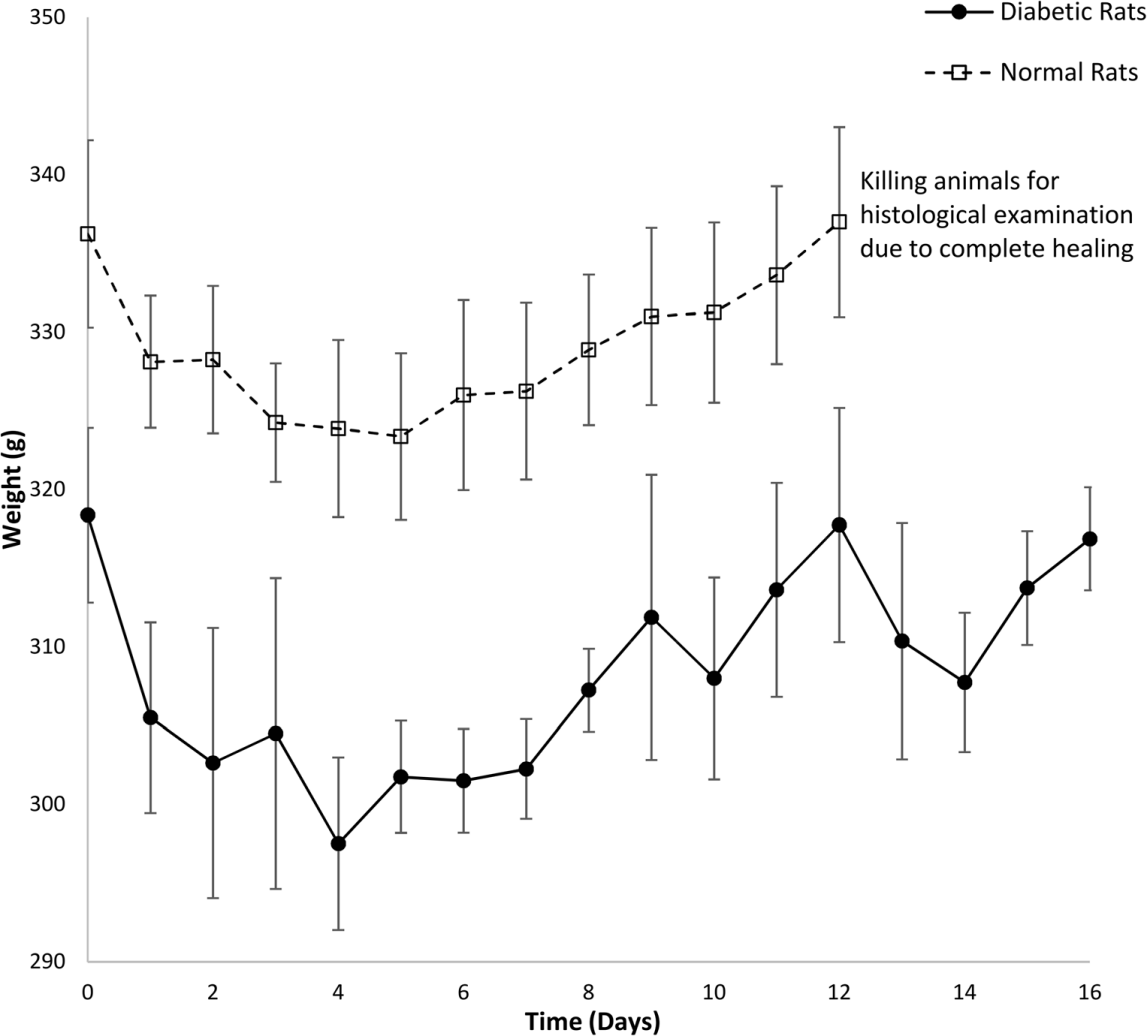
The average level of weight of eight diabetic and normal rats during 16 days. Results are mean ± SEM (*n* = 8)

In the non-diabetic group, body weight did not change significantly throughout the experiment, from 336 g at day 0 to 337 g at day 12 (Fig. 5). The average weight of eight diabetic rats was initially 318 g at day 0 and then significantly decreased to the minimum 298 g at day 4 (*p* < 0.05). As is shown in Fig. 5, diabetic rats started to restore body weight gradually during the healing process (316.87 g at day 16—end of experiment). This reduction arises due to proteolytic breakdown of structural protein into amino acids [29], followed by oxidation as cells fail to absorb blood glucose to be utilised as a metabolic energy source. Hepatic glycogenolysis and lipolysis contribute further to the reduction in weight in the diabetic cohort [30]. No complications were reported in any animals before and during surgery. There were no deaths prior to euthanasia. Daily clinical evaluations showed adequate recovery rate, maintenance status, presence of physical activity, provision of food and water intake in all groups.

### Wound healing in non-diabetic groups

In this work, the effect of acute diabetes on wound healing was investigated by comparing healing rate in non-diabetic and diabetic groups. Insulin was presented in two forms, namely encapsulated within a NP (F2) or as the free form. The role of insulin in wound repair follows several possible mechanisms [2]. Generally, insulin receptors belong to tyrosine kinase type, which are located on the surface of most cell types. Importantly, this distribution includes keratinocytes and fibroblasts that undertake key functions in wound healing [10]. Insulin has a direct proliferator and migratory effect through the activation of the PI3K-Akt pathway, and activates Rac1, a small GTPase, as a molecule stimulated downstream of PI3K-Akt [31]. This activation leads to translocation of Rac1 to the plasma membrane, followed by activation of Rac1 substrate, the integrin α3 and the extracellular matrix molecule laminin332 [32]. The effects of insulin on keratinocyte migration indicates that insulin-accelerated wound healing involves increased expression of the integrin α3β1 in keratinocytes as well as an increase in the levels of laminin 332 (LN332), which plays an important role in mediating keratinocyte polarity and cell migration [2].

**Fig. 6:**
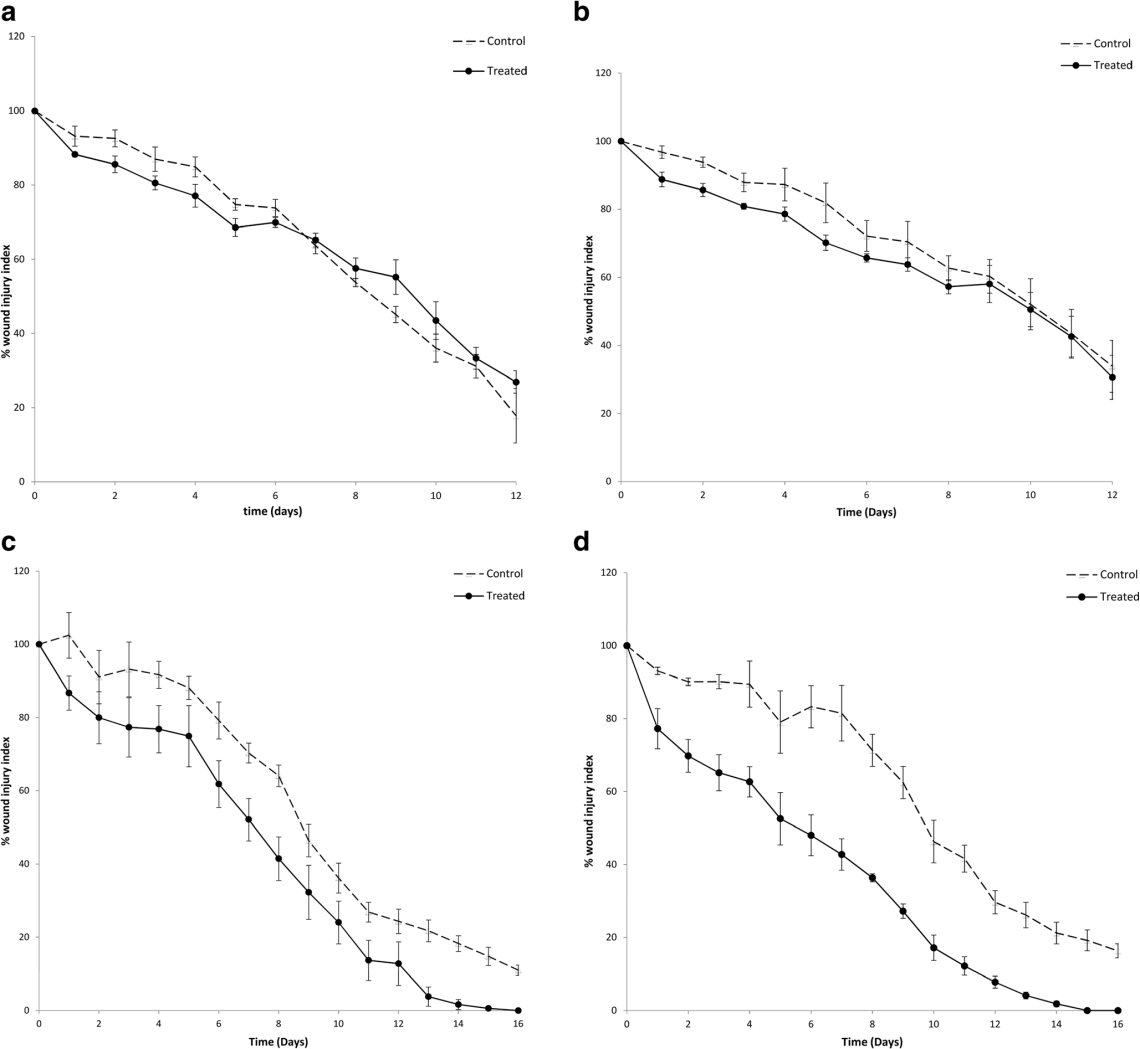
Percent wound injury index of **a** group non-DB-H, **b** group non-DB-NP, **c** group DB-H and **d** group DB-NP. Results show mean ± SME (*n* = 4). Paired *t* test results showed the statistical data of percent injury index of control and treated wounds. Non-significant difference (*p* > 0.05) (**a**) and significant difference *(*p* < 0.001) (b–d)

There was no significant difference between the control wound treated with saline and wounds treated with free insulin-loaded PVA-borate hydrogels for the non-diabetic group (Fig. 6a). Importantly, insulin encapsulated within PLGA NP did show a significant difference (*p* < 0.001) between the control and treated incisions of the non-diabetic rats (Fig. 6b). Delivery of insulin in a nanoparticulate carrier opens up the opportunity for drug deposition in the cytosol of keratinocytes, mediated by translocation passively through the cell membrane and active cellular uptake via endocytic pathways [33]. Findings in this study demonstrate that nanoencapsulation is a more efficient approach in reducing the wound area over time, when compared to direct exposure of the free drug. This effect was particularly evident (Fig. 6b) during the first 9 days of wound repair as no significant difference was observed in wound injury index between control and treated groups the study conclusion (days 10–12). Moreover, the addition of PEG to the NP formulations is expected to assist NP uptake due to its effect on reducing particle size [34]. However, further work is required to investigate how PEG affects cell uptake in the wound bed as published findings would support a conflicting reduction in cell uptake, particularly in reticulo-endothelial tissues.

### Wound healing in diabetic groups

The period needed for complete wound healing in the diabetic groups extended to 16 days (Fig. 6c, d). This was an expected finding, as diabetes impairs vascular flow, leading to poor tissue oxygenation and chronic hypoxia, all of which impair healing [35]. Data in Fig. 6c, d showed a significant difference (*p* < 0.001) in the percentage wound injury index observed between human insulin (free or formulated as NP) and their control wounds. This difference between treated and control groups was greater for the insulin-loaded NP group (DB-NP). For example, at day 10, the percentage wound injury index for the wound treated with insulin NP (F2) was 17.16% and its control was 46.31% (Fig. 6d), whereas for the wound treated with free insulin, it was 24.04% and its control was 36.20% (Fig. 6c).

**Fig. 7:**
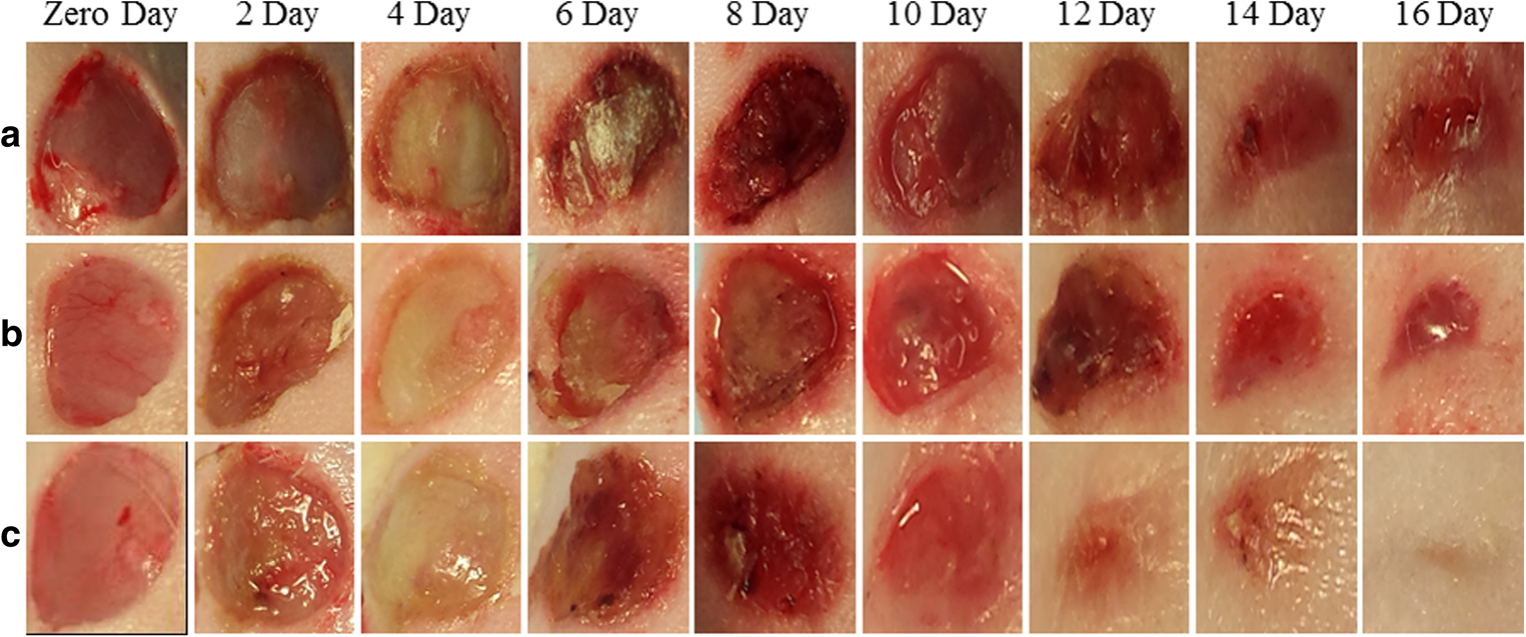
Macroscopic images of wound area showing re-epithelialisation stages. The wound area was measured at successive days till day (16) post-wound, (**a** control diabetic), (**b** DB-H) and (**c** DB-NP)

The development of skin wounds in all animals showed oozing and formation of a delicate crust up until the 8th and 12th day for DB-NP and DB-H groups, respectively (Fig. 7). Then, the crust thickened and began to detach as the healing process progressed. On the second post-operative day, an inflammatory process was observed, presenting congestion, hyperemia and necrosis. It is clear from images in Fig. 7 that slough started to appear in wound treated with insulin-loaded NP at Day 2 (row C), whereas for the control and a wound treated with free insulin, it appeared at day 4. Slough is a type of necrotic tissue, separating itself from the wound site. Sloughy tissue is non-viable, often white or yellow in colour. Once formed, slough detached following continuous application of treatment and epidermal cells began to be renewed in preparation for healing. Earlier slough appearance was considered a faster tissue response leading to earlier creation of a healed epidermal layer. Skin on the backs of the rats showed a pattern of free subcutaneous and elastic loose tissue.

### Histological evaluation

**Fig. 8:**
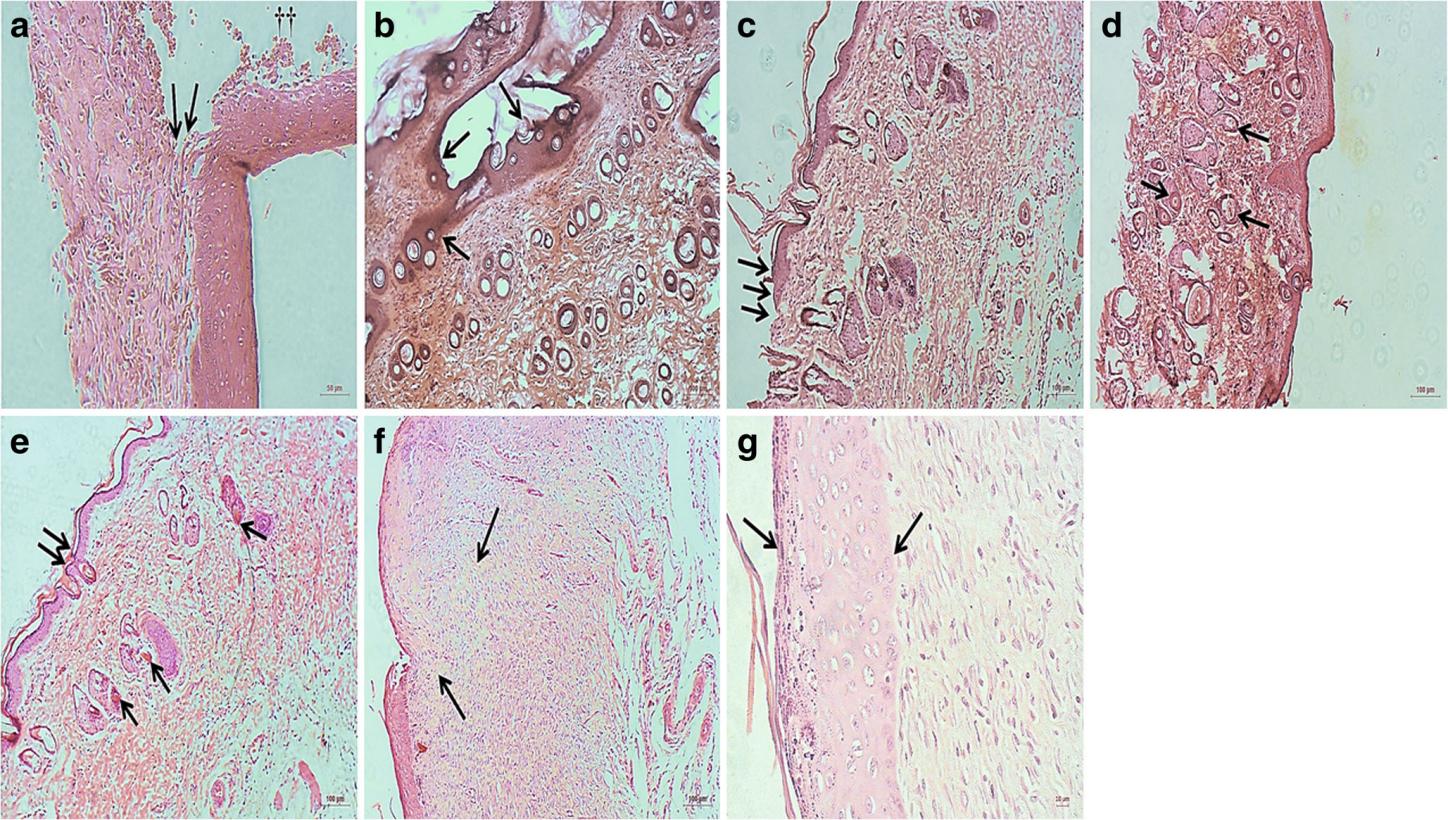
Haematoxylin and eosin-stained sections of cutaneous wounds on day 16 post-wounding. **a** Control wound from a diabetic rat treated with saline showing the presence of presence of scab tissue (arrows) and surgical threads (dagger). **b** Control wound from a diabetic rat showing localised tissue destruction, oedema and exudation (arrows). **c**, **d** Treated wounds in the DB-H group, showing irregular epidermis morphology (triple arrows) and the presence of hair follicles of different diameters in the dermis. The presence of hair follicles indicates that the tissue is healthy and growing normally. **e**–**g** Treated wound in the DB-NP group. **e** Regular epidermal morphology evident (double arrow) and increased angiogenesis in the dermis (single arrows) and formation of granulation tissue (arrow). **f** Localised increase in collagen fibres (arrows). **g** Intact epidermal layer with the arrows marking the lower and upper edges. Magnification bars = 100 μm (**b**–**f**), 50 μm (**a**) and 10 μm (**g**)

Histological visualisation confirmed the tissue repair findings observed macroscopically in animals treated with free insulin and insulin-loaded PLGA NP embedded in PVA-borate hydrogel. Images taken from tissue biopsies showed a rapid re-instatement of normal tissue structures following the application of topical insulin-loaded NP, in comparison to the control and free insulin, in the diabetic group (Fig. 8). Wounds in diabetic animals showed a delay in resolution, prolonged inflammation, angiogenesis and lower deposition of extracellular matrix. Topical insulin was able to restore the healing process in these diabetic animals, with better results observed following administrated of insulin-loaded NP when compared to free insulin. As shown in Fig. 8a, b, the inflammatory process was intense and diffused in the diabetic wound control. At day 16 post-wounding, a clear fibrinous tissue (scab) was still present (Fig. 8a) and localised tissue destruction was observed (Fig. 8b). In diabetic wounds treated with free insulin, a delay in healing was evident, as epidermal morphology was irregular (Fig. 8c) and the structure of dermal layer was impaired (Fig. 8d). Histological images of diabetic wounds treated with the insulin-loaded nanoparticulate carrier demonstrated a reduction in the inflammatory process, characterised by increased angiogenesis, formation of granulation tissue, completely reconstructed epidermis and collagen deposition (Fig. 8e–g).

## Conclusion

An obstacle that restrict the topical application of insulin is the lack of a suitable carrier that can deliver insulin reliably to the wound bed at a controllable rate. The design of a sophisticated delivery system, such as insulin-loaded particulate system in an optimised vehicle (PVA-borate hydrogel) was shown to be a promising strategy to ensure controlled delivery into wound environments [2]. The nanoparticulate formulation described in this work was stable and achieved useful levels of drug loading [12]. Wounds induced on rats and treated topically with insulin-loaded PLGA NP healed quicker than controls exposed to no insulin. Importantly, the findings of this work demonstrated the effectiveness of the nanoencapsulated delivery approach, in both diabetic and non-diabetic subjects. These findings were confirmed by histological evidence of angiogenesis and formation of granulation tissue. The importance of modifying the nanoparticulate formulation, such as addition of PEG, was shown to affect properties, such as size and drug loading, but further work is needed to verify the effect on endocytic cellular uptake and interaction with the secondary delivery vehicle. The findings highlight the potential of improved wound management using insulin-loaded colloidal carriers in structured hydrogel vehicles.
